# An automatic glaucoma grading method based on attention mechanism and EfficientNet-B3 network

**DOI:** 10.1371/journal.pone.0296229

**Published:** 2024-08-16

**Authors:** Xu Zhang, Fuji Lai, Weisi Chen, Chengyuan Yu

**Affiliations:** 1 School of Software Engineering, Xiamen University of Technology, Xiamen, China; 2 School of Computer and Information Engineering, Xiamen University of Technology, Xiamen, China; 3 School of Computer and Information Engineering, Jiangxi Agricultural University, Nanchang, China; South China University of Technology, CHINA

## Abstract

Glaucoma infection is rapidly spreading globally and the number of glaucoma patients is expected to exceed 110 million by 2040. Early identification and detection of glaucoma is particularly important as it can easily lead to irreversible vision damage or even blindness if not treated with intervention in the early stages. Deep learning has attracted much attention in the field of computer vision and has been widely studied especially in the recognition and diagnosis of ophthalmic diseases. It is challenging to efficiently extract effective features for accurate grading of glaucoma in a limited dataset. Currently, in glaucoma recognition algorithms, 2D fundus images are mainly used to automatically identify the disease or not, but do not distinguish between early or late stages; however, in clinical practice, the treatment of early and late glaucoma is not the same, so it is more important to proceed to achieve accurate grading of glaucoma. This study uses a private dataset containing modal data, 2D fundus images, and 3D-OCT scanner images, to extract the effective features therein to achieve an accurate triple classification (normal, early, and moderately advanced) for optimal performance on various measures. In view of this, this paper proposes an automatic glaucoma classification method based on the attention mechanism and EfficientNetB3 network. The EfficientNetB3 network and ResNet34 network are built to extract and fuse 2D fundus images and 3D-OCT scanner images, respectively, to achieve accurate classification. The proposed auto-classification method minimizes feature redundancy while improving classification accuracy, and incorporates an attention mechanism in the two-branch model, which enables the convolutional neural network to focus its attention on the main features of the eye and discard the meaningless black background region in the image to improve the performance of the model. The auto-classification method combined with the cross-entropy function achieves the highest accuracy up to 97.83%. Since the proposed automatic grading method is effective and ensures reliable decision-making for glaucoma screening, it can be used as a second opinion tool by doctors, which can greatly reduce missed diagnosis and misdiagnosis by doctors, and buy more time for patient’s treatment.

## 1. Introduction

Glaucoma, known as the "silent thief of sight", was recognized as an eye disease in the early 17th century. It begins with damage to the optic nerve due to increased pressure in the eye, called intraocular pressure [1]. The longer the increase in intraocular pressure persists, the more severe the damage to visual function. If left untreated, glaucoma can lead to irreversible visual impairment and even blindness.

According to the latest data, by 2021, approximately 76 million people were suffering from glaucoma worldwide. The number of glaucoma patients in China is approximately 22 million, of which approximately 5.7 million are blind. Therefore, it is imperative to protect your eyesight. Common glaucoma symptoms include sudden loss of vision, severe eye pain, blurred vision, and eye redness. Glaucoma predominantly affects middle-aged and older populations, especially those over the age of 40. Therefore, age is a common risk factor for glaucoma. Early detection and treatment are essential to preventing vision problems caused by glaucoma [2]. Ophthalmologists use different examination methods to examine patients, such as ophthalmoscopy, tonometer, and visual field measurement using a visual field metre for comprehensive glaucoma diagnosis. The ophthalmoscope examination method is used to check the colour and shape of the object, while the tonometer is used to measure internal intraocular pressure. The visual field metre examination is used to analyse the size of the visual field [3]. The complexity of fundus images and the time-consuming and subjective judgement of doctors interfere with traditional manual recognition methods, thus leading to misdiagnosis and omission of glaucoma. Therefore, introducing computer-aided diagnostic (CAD) systems as an important aid to physicians has become particularly urgent. CAD systems play an indispensable role in ensuring an accurate, reliable, and rapid diagnosis of glaucoma [4]. Glaucoma CAD systems can use retinal fundus images as input and classify them as "abnormal" or "normal" by extracting information from multiple feature types. This system can effectively assist doctors in making rapid and accurate glaucoma diagnoses [5]. The Glaucoma CAD system serves a key support role in physician diagnosis, significantly improves physician productivity, reduces physician workload, and greatly reduces the risk of misclassification. The system provides doctors with reliable and objective data, enabling medical teams to process large volumes of fundus images more efficiently and ensuring that patients receive timely and accurate diagnosis and treatment [6]. Artificial intelligence technology can automatically identify glaucoma without much a priori knowledge, so many scholars have attempted to use artificial intelligence technology for patient screening of fundus images. Deep CNNs have proven to be an efficient AI-based tool in identifying clinically significant features from retinal fundus images [7–10].

To obtain a more reliable automatic glaucoma grading algorithm, this project proposes an automatic glaucoma grading method based on the attention mechanism and EfficientNet-B3 network and proposes using two modal data, a 2D fundus image, and a 3D-OCT scanner for model training, testing, and validation to automatically identify glaucoma to achieve higher identification accuracy.

Marcos et al. [11] used a convolutional neural network (CNN) for optic disc segmentation, a step that helps to accurately locate and extract optic disc regions in fundus images. Next, they used advanced image processing techniques to remove nonoptic disc tissues, such as blood vessels, to highlight the features of the optic disc more prominently. The team then focused on extracting textural features of the optic disc region that are important for diagnosing and classifying ophthalmic diseases. Finally, they used these features for classification tasks, which provide reliable support for clinical diagnosis through learning and inference by convolutional neural networks. Raghavendra et al. [12] proposed a novel computer-aided diagnosis (CAD) system for accurately detecting glaucoma using deep learning techniques and designed an 18-layer convolutional neural network. After effective training to extract and then test the features for classification, the system provides a good solution for the early and fast-assisted diagnosis of glaucoma patients. Chai et al. [13] proposed a multibranch neural network (MB-NN) model, which is utilized to adequately extract the deep features from the image and is combined with medical domain knowledge to achieve classification. Balasubramanian et al. [14] attempted feature extraction via a histogram of orientation gradients (HOG) combined with a support vector machine (SVM) for glaucoma classification. However, this method requires tedious preprocessing steps and performs poorly in terms of accuracy. In research on hybrid structures based on structure splicing, Carion et al. [15] proposed DETR, which uses the ResNet backbone network to extract compact image feature representations to generate low-resolution and high-quality feature maps, which effectively reduces the size of the input transformer image scale and improves the speed and performance of the model. Ding Pengli et al. [16] proposed CompactNet, a compact neural network based on a compact neural network, to identify and classify retinal images; however, due to the limited experimental samples, the network did not sufficiently extract the relevant features in the training process, so the classification accuracy was not high. Hongjie Gao [17] proposed an algorithm for the vascular segmentation of fundus images with an improved U-shaped network. The algorithm uses the idea of the residual network to change the traditional serial connection method of convolutional layers to the residual mapping phase superposition method and adds batch normalization and a PReLU activation function between the convolutional layers to optimize the network. The algorithm was tested on the DRIVE and CHASE_DB1 fundus databases and compared to the best mainstream algorithms in terms of accuracy, sensitivity, and AUC, which improved by 2.47%, 0.21%, and 0.35%, respectively, on average. Huang Yuankang et al. [18] proposed a method based on Markov random field theory for extracting the optic disc contour of fundus images. Meanwhile, they used the Euclidean distance and correlation coefficient identification method based on the ISNT law for classifying glaucoma fundus images. However, this method requires manual assistance to complete, which is less efficient and less automatic. Panming Li [19] proposed a two-stage automatic SS point localization algorithm based on Gaussian heatmap regression and deep reinforcement learning. Optimal classification performance was achieved in a classification model based on SE-ResNet18. Law Kumar Singh et al. [20–23] completed several studies in which they identified convolutional neural network (CNN) models as the best performing deep learning model for automated glaucoma detection by comparing different such as Inception-ResNet-v2 and Xception. They further used bio-heuristic algorithms to optimise the feature selection process and proposed two effective two-layer feature selection methods (BAT-MLC-BCS-MLC and BCS-MLC-PSO-MLC), as well as Gravitational search optimization algorithm (GSOA), which reduce the number of number of features while maintaining high accuracy detection capability. In addition, they proposed an Emperor penguin optimisation algorithm- and bacterial foraging optimization algorithm-based novel feature selection approach. this approach reduces the number of features by balancing global and local search, improves the accuracy of the classifier, and achieves an accuracy of 0.95410 when used in conjunction with the Random Forest classifier. These studies provide innovative solutions to practical problems in the field of medical diagnosis. The research of Sadaqat ur Rehman et al. [24–27] focuses on improving the efficiency and performance of Convolutional Neural Networks (ConvNet), especially in the unsupervised pre-training phase. They proposed the CSFL (Convolution Sparse Filter Learning) algorithm, which is a novel unsupervised CNN method that improves the efficiency of visual pattern classification by using a sparsity function to measure the sparsity of the features so that more discriminative features can be learned. In addition, they proposed the MRPROP algorithm, an improved RPROP algorithm for optimizing the training of CNNs. MRPROP prevents overfitting by introducing a tolerant band, which is combined with the concept of global optimum for weight updating, allowing the network to adjust the weights more quickly and accurately. The application of unsupervised pre-trained sparse filters to facial recognition tasks is also explored, demonstrating that this approach achieves better performance and faster convergence during training compared to random filters. Subsequently, they also explored the application of unsupervised pre-trained sparse filters in facial recognition tasks, demonstrating that this approach achieves better performance and faster convergence during training compared to random filters. Jahanzaib Latif et al. [28, 29] proposed the ODGNet model, a two-stage deep learning model combining visual saliency models and deep learning techniques for automatic localization of the optic disc and glaucoma classification. ODGNet was evaluated on several public retinal datasets, with the ORIGA dataset achieving a diagnostic accuracy of 95.75% and a 97.85% AUC value, showing high accuracy and efficiency. In addition, they explored the application of Inception V-3 model-based transfer learning in glaucoma detection, using pre-trained CNN models to improve classification accuracy and address the problem of insufficient datasets. Despite the high accuracy achieved on the validation set, the large number of model parameters and small sample data size may lead to overfitting and affect the generalization ability of the model. These studies provide new methods and insights for automated glaucoma detection.

Generally, at present, domestic and foreign research teams mainly use traditional neural networks for glaucoma recognition research, which has the following defects. First, its dataset only adopts the most common 2D fundus image with a single modality, focusing on normal and glaucomatous dichotomous classification. Second, it fails to account for the large and meaningless black background in the 2D fundus image, which makes its performance poor. Finally, the above methods have scope for improvement in terms of accuracy, kappa value, recall, and F1 value. Rate and F1 value have room for improvement. However, this experimental method adopts two modal data, a 2D fundus colour photo and a 3D OCT scanner, as the experimental dataset, which achieves multimodality and more accurate image feature extraction. Second, this experiment fully analyses the characteristics of the fundus images and uses the attention mechanism to discard the meaningless black background so that the convolutional neural network focuses more on the main features of the eye, thus improving the recognition and grading performance. Moreover, this experimental goal is to achieve higher accuracy, kappa value, recall, and F1 value, so the use of the EfficientNet-B3 network, which is relatively new, can more accurately achieve automatic glaucoma grading.

## 2. Data and methodology

### 2.1 Dataset

**Fig 1:**
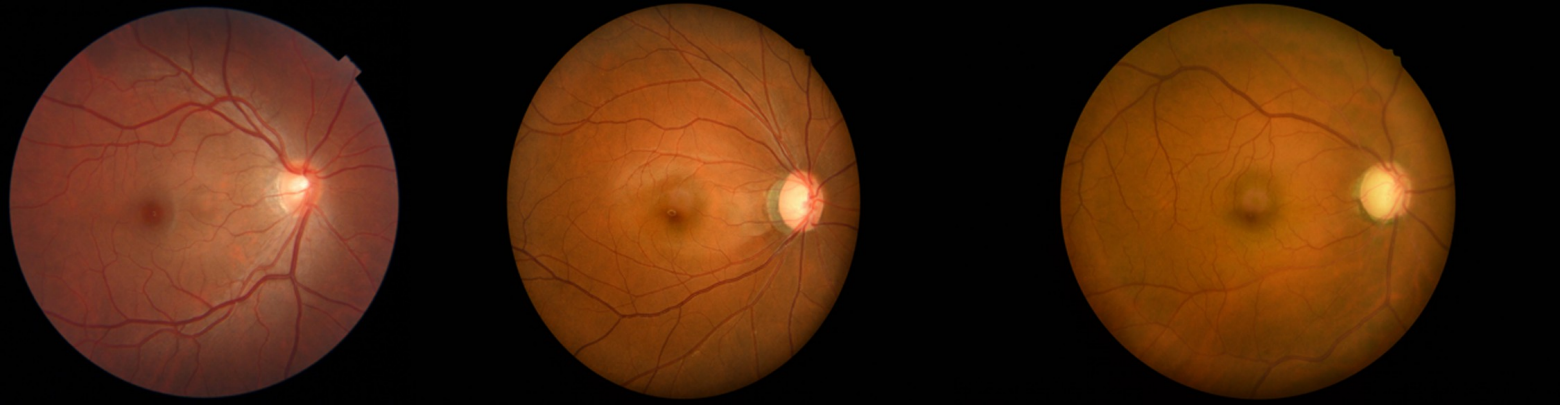
2D fundus images.

**Fig 2:**
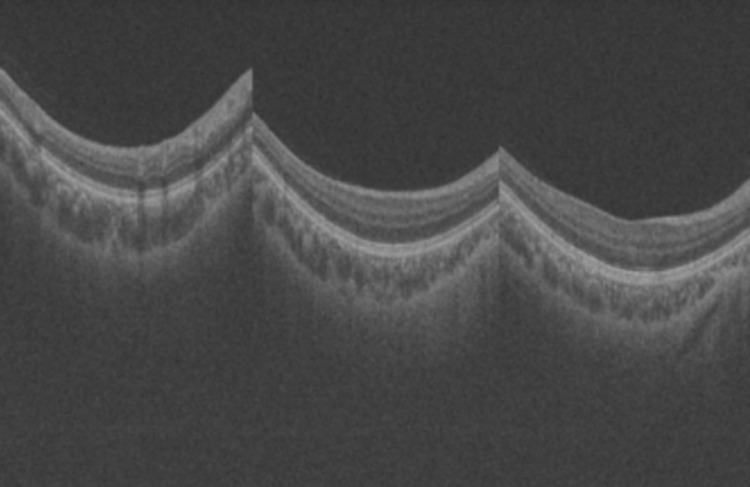
3D-OCT scanner images.

The dataset used in this experiment was provided by Zhongshan Ophthalmology Centre, Sun Yat-sen University, Guangzhou, China, which contains 200 data pairs of two clinical modality images: 100 pairs in the training set and 100 pairs in the test set. The two modalities are 2D fundus image colour photographs and 3D optical coherence tomography (OCT), commonly performed in clinical fundus examinations. For deep learning algorithms, 100 training data pairs are small samples, so the recognition model proposed in this experiment is suitable for training on small sample datasets. The reason why these two modalities are characterised is that 3D-OCT images can reveal changes in the thickness of the RNFL, which is a key indicator for the early diagnosis of glaucoma, and 3D-OCT images are also capable of detecting microstructural changes in the retina and optic nerve head, which is an important piece of information that may be difficult to detect in 2D fundus images. Therefore, the use of 3D-OCT scanner images in conjunction will not only help to better differentiate whether a patient has glaucoma or not, but also better define the severity of glaucoma, which belongs to a more refined classification, and therefore will be superior to the unimodal approach in terms of accuracy. Fig 1 shows some 2D fundus images, which will be used for EfficientNet-B3 network training. Fig 2 shows some 3D-OCT images, which will be used for ResNet34 network training after convolution.

### 2.2 Data preprocessing

In this experiment, data enhancement methods such as flipping (horizontal + vertical), adding noise, random rotation, random flip, random change in brightness, random change in contrast, random change in saturation, cropping, scaling/stretching, and blurring are used. By applying these approaches individually or in combination, the dataset can be processed to increase the quantity of data, capture more image features, and enable the model to see more data variations, improving the model’s generalizability. The use of these data enhancement modalities can effectively address the lack of data volume and mitigate the overfitting problem of the model, as well as improve the model’s ability to adapt to new data, which can help enhance the model’s ability to perform in the glaucoma autoclassification task.

### 2.3 EfficientNet-B3 network training model

**Fig 3:**
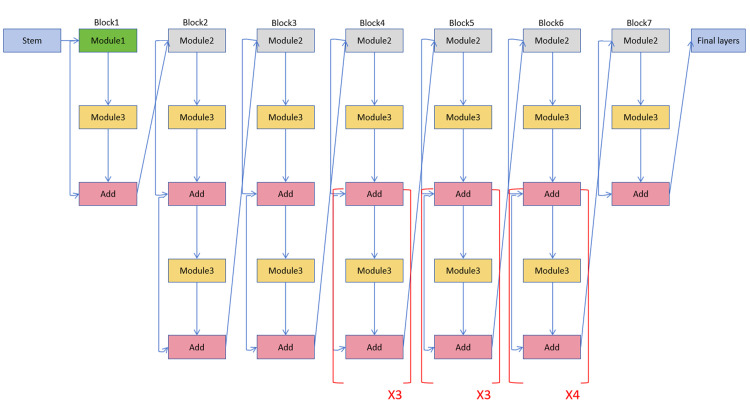
EfficientNet-B3 network structure.

EfficientNet-B3 is a network model with unique features whose design benefits from the experience of other good neural networks. The network model contains a residual structure that not only deepens the depth of the network but also makes feature extraction more accurate and efficient. In addition, it allows the flexibility of adjusting the number of feature layers in each layer to achieve more layers of feature extraction, thus enhancing the width of the network. In addition, EfficientNet-B3 can learn and express information from richer data by enlarging the input image solution resolution, which can help enhance model precision. Overall, EfficientNet-B3 is an efficient and flexible network model that draws on the design of many excellent neural networks, making it perform well in a variety of tasks. Fig 3 is a schematic structural diagram of the EfficientNet-B3 network.

### 2.4 ResNet34 network model

In deep learning, deep neural networks are a very effective model. However, as the network layers increase, some problems arise, such as gradient vanishing and gradient explosion. These problems make training deep networks very difficult. Therefore, to overcome these problems, there are some solutions, one of which is ResNet, which is a kind of residual neural network. The fundamental concept is to construct a deep network by adding "residual blocks". The core idea is to introduce cross-layer connections in the network so that the information can be passed directly from the front layer to the back layer. Such cross-layer connections can relieve the gradient vanishing and gradient exploding problems effectively, making training deep networks easier. With ResNet, networks can become deeper without causing performance degradation or training difficulties. This makes ResNet an essential breakthrough in the deep learning field, helping to solve more complex tasks and handle larger data. As a result, ResNet has been widely used in deep learning research and practical applications.

**Fig 4:**
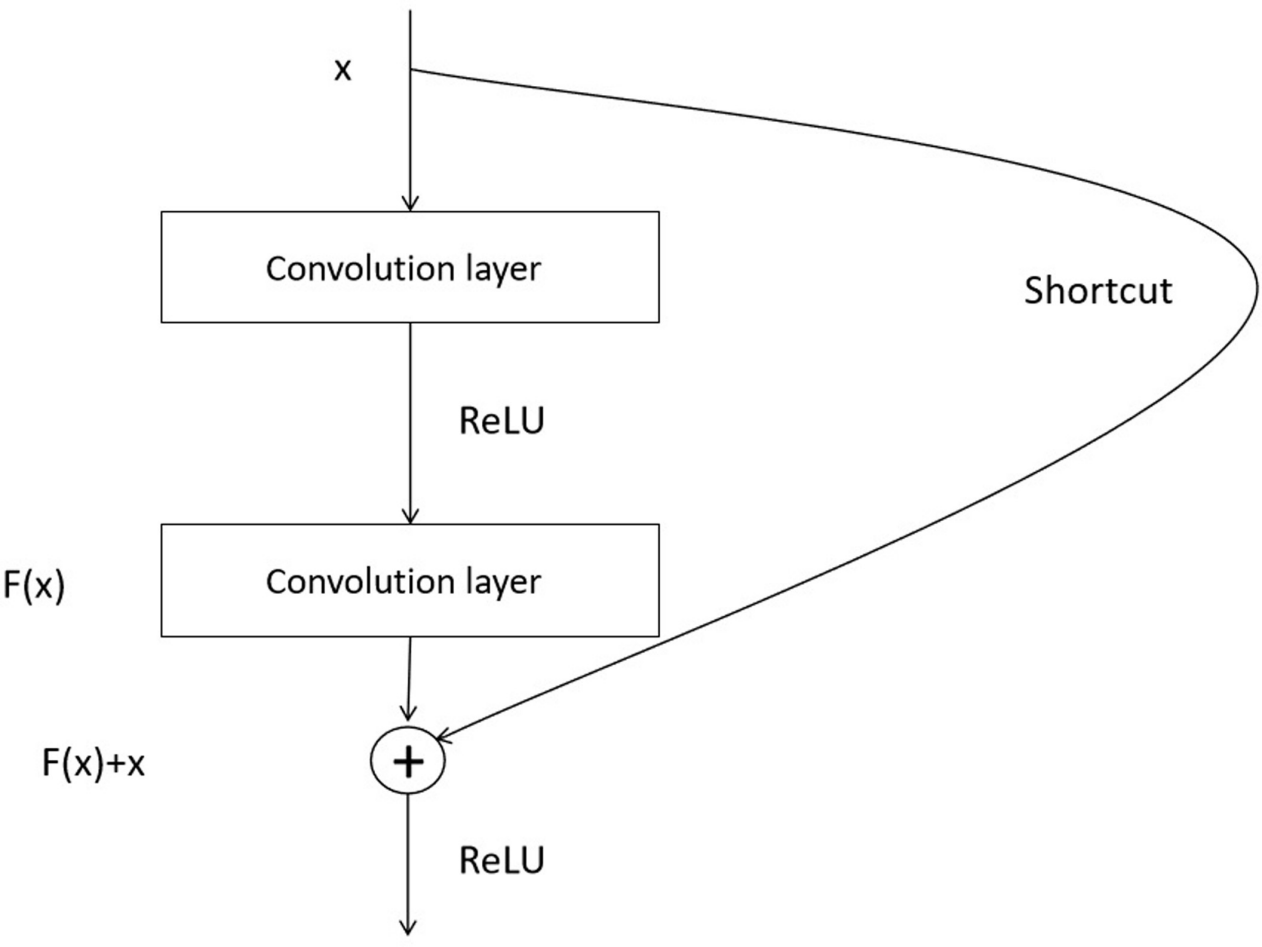
Residual block.

ResNet34 is a relatively concise ResNet structure containing 34 convolutional layers and 18 residual blocks. It is inspired by solving the vanishing and exploding gradient problems in deep neural networks. First, the input layer, ResNet34, is an ordinary convolutional layer including 64 convolutional kernels, each of which is 7×7 in size, with a stride of 2 and padding of 3. The main purpose of this layer is to cut the input image in half in terms of size and to extend the lower-level features out of the image. Next is the residual block. ResNet34 has a total of 18 residual blocks. Every residual block is composed of two convolutional layers of size 3 × 3 and a cross-layer connection. For the first convolutional layer, the step size is 1, and the padding is 1. The second convolutional layer also has a step size of 1 and a padding of 1. The cross-layer connection enables the outer output of the former layer to be directly appended to the input of the latter layer, which preserves the message of the earlier layer and passes it on to the latter layer. This design helps in information transfer and mitigates the vanishing and exploding gradient problems. Thus, ResNet34 effectively addresses some of the difficulties in training deep neural networks by introducing residual blocks and cross-layer connections. This design allows the network to be deeper, easier to train and to achieve excellent performance with relatively few parameters. Furthermore, there is a global average pooling layer, which is added following the latest residual block of ResNet34. The role of this layer is to average pool the output of the last residual block to obtain a global feature. Global average pooling is an operation that compresses the entire feature map into a single value. Through this operation, the network can obtain comprehensive information about the whole image and thus better understand the overall semantics. Finally, there is a full-connectivity layer, which is inserted following the global average pooling layer. The role of this layer is to map global features to category scores. The full-connectivity layer is typically used for performing classification tasks, where associations are established between the extracted features and the categories to obtain scores or probabilities for different categories. Ultimately, the model makes classification decisions based on these scores or probabilities. With the global average pooling layer and the fully connected layer, ResNet34 can map image features to the final category scores to perform tasks such as image classification. Introducing global average pooling and fully connected layers gives ResNet34 a powerful classification capability and allows it to perform well in a variety of image recognition problems. Fig 4 is a schematic diagram of the Residual block structure.

### 2.5 Attention mechanism

Attentional mechanisms have achieved significant success in computer vision tasks but have been less frequently applied in glaucoma recognition and automatic grading tasks. This may be because the job involves complex medical images, small datasets, and difficult labelling, while the attention mechanism requires larger datasets and expert support. However, applying attention mechanisms in glaucoma recognition and automatic grading is still worth exploring as profound learning techniques advance and datasets increase.

The attentional mechanism works by focusing on the most salient parts of the characteristics that are extracted by the deep neural network, thereby eliminating redundant information from the visual task. This mechanism is usually implemented by embedding an attention map into the neural network. The attention mechanism allows the neural network to automatically learn and select the most critical regions and features in the image while ignoring the unimportant parts. In this way, the network can focus on meaningful information more effectively, improving task performance and efficiency. The fundus image in this experimental dataset contains many pointless black background areas, which can result in many redundant features, so this experiment adds an attention mechanism to the feature extraction process so that the convolutional neural network focuses more on the main features of the eyes, thus improving the performance of the glaucoma autoclassification model. The Attention Module of this method consists of two sub-modules, namely the Channel Attention Module and the Spatial Attention Module. The Channel Attention Module is used to Assign weights to the feature maps of each channel to improve the response of important channels. The Spatial Attention Module is used to assign weights to the feature maps of each spatial location to improve the response of important locations. By combining the two, the weighting of important features can be enhanced, thus enhancing the modelling capability of spatial and channel information and improving the performance of the model. Li Liu et al. [30] proposed an attention-based convolutional neural network (AG-CNN) for glaucoma detection, their proposed attention mechanism focuses more on how to predict the attention maps through weakly supervised learning, and some of their training images require ophthalmologists’ labeled attention maps, which requires doctors’ time and effort. Most of the existing studies adopt this approach, and the proposed attention mechanism is not as effective when it is validated in 3D-OCT scanner images. In this paper, the combination of channel and spatial attention modules is a more general approach to achieve finer-grained control, and therefore performs better when utilized in the two branch networks in this paper.

### 2.6 Loss function

Since automatic glaucoma classification is a three-classification task, the softmax function plus the cross-entropy loss function is adopted as the loss function in this paper. Using the softmax function can restrict the output between 0 and 1, and the sum of the probabilities of each sample falling into each category is just 1. The cross-entropy loss value is calculated by the formula: $\text{loss}=-\sum_{c=1}^{M}y_{c}\log\left(p_{c}\right)$, where M is the number of categories, *y*_*c*_ is equal to 0 or 1, 1 if the predicted category is the same as the sample labelling, 0 otherwise, and *p*_*c*_ is the probability that the sample belongs to category c.

Finally, the loss results of all the samples in the training set are summed to obtain the final total loss.

## 3. Results

### 3.1 Experimental environment

All the algorithms in this article are performed in the following hardware environment. GPU: TeslaV100, Memory: 32GB, CPU: 4 cores, implemented using the Python language based on the paddle deep learning framework.

### 3.2 Results and analyses

Since the time complexity of data loading is proportional to the size of the dataset, the time for both the ResNet34 network and the EfficientNetB3 network is O(N). To validate the usefulness of the data extension method, in this paper, we trained the EfficientNet-B3 model on the primary dataset and the extended dataset for 50 epochs. After training was completed, we evaluated the model using an independent test set and recorded the accuracy of the model. By comparing the performance of the models trained on the original and expanded datasets, we can conclude that the data expansion method has a positive effect on model performance. The results of the tests are displayed in Table 1.

**Table 1:** Comparison of the test results before and after expanding the data.

Dataset	Model	Epoch	Accuracy
Primary dataset	EfficientNet-B3	50	96.61%
Augment dataset	EfficientNet-B3	50	97.58%

Table 1 demonstrates the effect of data expansion on model performance. The accuracy of the EfficientNet-B3 model on the original dataset is 96.61%, whereas, through data expansion, the model accuracy is increased to 97.58%, which is an improvement of 0.97%. This result shows that model accuracy and performance are significantly enhanced by data enlargement.

To continue to enhance the model’s efficiency, this paper adopts an effective method for removing unnecessary redundant features in the convolutional neural network by applying an attention mechanism to the EfficientNet-B3 network structure, which can significantly improve the performance of glaucoma autograding. GoogLeNet [31], for the first time, proposed the use of convolutional kernels of multiple sizes for simultaneous feature extraction, and this method is known as the inception module. By introducing the inception module, GoogLeNet can extract a wide range of features at different scales and levels, thus increasing the network width and allowing it to better capture information at different scales. However, ResNet has successfully strengthened the delivery of gradient information across the network through feature reuse. This result is due to the introduction of residual blocks and cross-layer connectivity. This design allows information to be passed in jumps, mitigating the problems of vanishing and exploding gradients while also allowing the network to become deeper and easier to train. These two network architectures have excellent performance in many computer vision tasks, so GoogLeNet and ResNet are constructed as baseline models for comparison in this experimental work. In this experiment, GoogLeNet, ResNet, and the presented model are tested on the extended dataset with 200 training epochs. The tested model is confirmed on the test set, and the accuracy, kappa value, recall, and F1 value of the model are calculated. The results of the tests are displayed in Table 2.

**Table 2:** Comparing the results of tests with the baseline model.

Model	Accuracy	kappa	Recall	F1-score
GoogLeNet	94.79%	0.9714	94.32%	95.91%
ResNet	96.84%	0.9802	96.69%	96.11%
State-of-the-art model(YOLOv7)	97.70%	0.9877	96.81%	96.36%
EfficientNet-B3+ResNet34	**97.83%**	**0.9911**	**97.24%**	**97.03%**

Table 2 demonstrates the results of the trials for the baseline models, in which the accuracy, kappa value, recall, and F1 values are 94.79%, 0.9714, 94.32%, and 95.91% for GoogLeNet, 96.84%, 0.9802, 96.69%, and 96.11% for ResNet and 97.70%, 0.9877, 96.81%, and 96.36% for State-of-the-art model(YOLOv7), whereas the values of accuracy, kappa value, recall and F1 value for EfficientNet-B3+ResNet34 are 97.83%, 0.9911, 97.24% and 97.03%, respectively. Indicators also remain better than current state-of-the-art model.

Compared with the baseline model, the accuracy, kappa value, recall, and F1 value of the EfficientNet-B3+ResNet34 proposed in this paper, which is built on the attention mechanism, are at the optimal level in all four metrics, since traditional baseline networks such as GoogleNet are trained and validated in public datasets, the learning rate relies on the researcher’s experience and requires a large number of experiments to obtain a better learning rate; the present method is paired with the use of the Adam optimizer, which is capable of adaptively adjusting the learning rate of each parameter to improve the model’s convergence speed and generalization ability. so it can be demonstrated that by using both modal data of 2D and 3D OCT scanners as the experimental dataset and using the EfficientNet-B3+ResNet34 network for glaucoma recognition and autograding, not only is the accuracy improved but its performance is also enhanced.

### 3.3 Ablation study

In order to verify the effectiveness of the attention mechanism module and hybrid model proposed in this paper in the glaucoma auto-classification task, the modules were added to the original ResNet34 network and EfficientNetB3 network for experiments, respectively. Table 3 shows the results of the ablation experiments.

**Table 3:** Results of ablation experiments.

Method	time consuming (h)	Accuracy
EfficientNetB3	1.6	95.31%
ResNet34	1.5	93.19%
EfficientNetB3+ ResNet34	2.8	97.22%
EfficientNetB3+ ResNet34+ Attention Mechanism	**1.3**	**97.83%**

From the results of the ablation experiments in Table 3, it can be seen that the combination of the EfficientNetB3 network and ResNet34 network with the attention mechanism has achieved the best results in terms of time consumption and accuracy metrics, thus proving the effectiveness of the combination of EfficientNetB3 network and ResNet34 network with the attention mechanism module proposed in this paper.

### 3.4 Comparison with the literature

To further highlight the value and contribution of the work in this article, the proposed model is analysed in comparison with the existing achievements in the literature. The results of the comparison are detailed in Table 4 below.

**Table 4:** Comparative results with the literature.

Method	Model	Accuracy
Balasubramanian et al [29]	HOG	96.08%
Ding Pong Li et al.	CompactNet	97.60%
Gao Hongjie et al.	Improved U-shaped network	97.69%
Proposed method	EfficientNet-B3+ResNet34	**97.83%**

Table 4 gives the results of the comparison between the work in this paper and the work in the literature, in which Balasubramanian et al. achieved an accuracy of 96.08% in glaucoma classification using histogram of orientation gradients (HOG) for feature extraction and combining it with support vector machines (SVMs), while Pongli Ding et al. used a compact neural network based on CompactNet, with an accuracy of 97.60%. Hongjie Gao et al. used an improved U-shaped network with an accuracy of 97.69%. In this paper, after the data enlargement process, the EfficientNet-B3+ResNet34 for the attention-based regime was intensively trained, and a model with excellent performance was obtained. On an independent testing set, the model demonstrated an accuracy as high as 97.83%, which fully proved the value of the model in the field of glaucoma automatic identification.

By comparing the current model with the most accurate glaucoma recognition rate, the accuracy of this experimental model for recognition and automatic grading is better than the current latest, most effective method, therefore demonstrating the validity of the work in this article.

## 4. Scope, limitation and future work

In this study, based on a private dataset, two branch networks, EfficientNetB3 network and ResNet34 network, were used to facilitate effective feature extraction of 2D fundus images and 3D-OCT scanner images, and finally accurately complete the automatic grading of normal, early, and intermediate-late glaucoma, which can not only help doctors to help patients to detect the presence or absence of glaucoma, but can also This not only helps doctors to help patients to detect the presence of glaucoma, but also helps them to identify the severity of glaucoma so as to treat the symptoms, which is of great significance for the protection of patients’ vision. In this work, the dataset was first augmented, a method that can improve the robustness of the model to some extent, but the effect is limited. In future work, the need to validate the present method on more datasets, even though the current publicly available dataset only has 2D fundus images and the accompanying 3D-OCT scanner images are difficult to obtain. In addition to this, there is a high demand for the quality of the dataset. Therefore, in the future, it is necessary to strengthen the cooperation with hospitals to obtain more high-quality private datasets and improve the generalisation ability of the model. The proposed methodological model can also still be further improved. For example, more complex features can be captured by adding more and wider layers by increasing the repetitive blocks (blocks) and sub-blocks (sub-blocks) of the EfficientNet network, although this may also result in a larger number of parameters and require higher computational costs, but all in all, the aim is to seek better performance and results. In the future, reference can also be made to the use of other networks. As a future direction, hyperparameter tuning and optimisation based on algorithms such as 3D Convolutional Neural Networks (3D CNN) could be used.

## 5. Conclusion

Glaucoma is the second most common blinding eye disease in the world. With the number of glaucoma patients increasing rapidly each year, early and effective detection is essential to prevent vision problems caused by glaucoma. Traditional glaucoma screening methods rely on doctors’ subjective judgement, which can easily lead to missed diagnoses and misdiagnoses. The use of artificial intelligence to help doctors diagnose glaucoma is an important topic in the medical field. In this study, we propose an automatic glaucoma grading method based on the attention mechanism and EfficientNetB3 network for identifying three states of glaucoma (normal, early, and intermediate-late) in 2D fundus images and 3D-OCT scanner images. The proposed method was subjected to various experiments on a private dataset. Firstly, to address the problem of small data samples, each sample in the dataset was expanded using data augmentation. In order to enhance the performance of the model, an attention mechanism is added to the branching network so that the model extracts effective features faster and improves the model performance. This study conducted several comparison experiments with the benchmark model and related studies, and the experimental results fully demonstrate the superiority of the model in terms of performance, accuracy, and other aspects, especially in terms of accuracy, up to 97.83%, which is better than other current algorithms in the same direction. Professional ophthalmologists can use the proposed method as a second opinion when diagnosing glaucoma. The method is easy to build the model and easy to use in terms of clinical implementation, and only the patient’s fundus image and scanned body image are required for its use. For doctors, this method is fast and reliable, which greatly improves their diagnostic efficiency, avoids misdiagnosis to the greatest extent possible, makes accurate judgement of the patient’s condition in a timely manner, intervenes in the treatment, and protects the patient’s eyesight.
